# Reversal by aqueous extracts of *Cistanche tubulosa* from behavioral deficits in Alzheimer’s disease-like rat model: relevance for amyloid deposition and central neurotransmitter function

**DOI:** 10.1186/1472-6882-14-202

**Published:** 2014-06-26

**Authors:** Chi-Rei Wu, Hang-Ching Lin, Muh-Hwan Su

**Affiliations:** 1 Department of Chinese Pharmaceutical Sciences and Chinese Medicine Resources, College of Pharmacy, China Medical University, No. 91, Hsieh Shih Road, Taichung, 40402, Taiwan; 2 School of Pharmacy, National Defense Medical Center, No. 161, Sec. 6, Minquan E. Rd., Neihu Dist., Taipei 11490, Taiwan; 3 Sinphar Pharmaceutical Co., Ltd., Sinphar Group (Taiwan), Research & Development Center, No. 84, Chung Shan Rd., Chung Shan Village, Tung-Shan Shine, I-Lan 26944, Taiwan

**Keywords:** *Cistanche tubulosa*, Amyloid β peptide 1-42, Morris water maze, Acetylcholine, Dopamine

## Abstract

**Background:**

*Cistanche tubulosa* (Schenk) R. Wight (CT) is commonly used to treat forgetfulness by traditional Chinese physicians. This study presents the ameliorating effects of CT extract which was quantified with three phenylpropanoid glycosides in Alzheimer’s disease (AD)-like rat model.

**Methods:**

Amyloid β peptide 1-42 (Aβ 1-42) intracisternally infused to rats by osmotic pump (Alzet 2002) was used as an AD-like rat model. The major pathological makers were measured including Aβ 1-42 immunohistochemical stain, behavioral tests (inhibitory avoidance task and Morris water maze) and central neurotransmitter functions.

**Results:**

Aβ 1-42 caused the cognitive deficits, the increase in the amyloid deposition and acetylcholinesterase activities, and the decrease in the levels of brain’s acetylcholine and dopamine. Daily administration of CT extract throughout Aβ 1-42 infusion periods ameliorated the cognitive deficits, decreased amyloid deposition and reversed cholinergic and hippocampal dopaminergic dysfunction caused by Aβ 1-42. Donepezil also ameliorated the cognitive dysfunction, but only blocked the amyloid deposition and cholinergic dysfunction caused by Aβ 1-42.

**Conclusions:**

We suggest that CT extract, containing enough echinacoside and acteoside, ameliorated the cognitive dysfunction caused by Aβ 1-42 via blocking amyloid deposition, reversing cholinergic and hippocampal dopaminergic neuronal function.

## Background

Alzheimer’s disease (AD), a progressive neurodegenerative disorder, is characterized by cognitive deficits and three neuropathological hallmarks: neuronal loss, senile plaques and neurofibrillary tangles [1]. The major pathogenesis of AD includes the cholinergic hypothesis and the amyloid cascade hypothesis [2]. The core feature of amyloid cascade hypothesis is the formation of the byproduct amyloid β peptide (Aβ) from amyloid precursor protein via amyloidogenic pathway [1,2]. When Aβ is deposited in the brain, it is associated with cerebral neuronal loss, particularly the degeneration of cholinergic neuronal circuits in the basal forebrain (the cholinergic hypothesis) [2,3]. A series of studies suggested that the intracisternal infusion of Aβ 1-42 into rats was primarily deposited in the frontal cortex and the hippocampus, causing memory deficits in behavioral tasks including inhibitory avoidance task and Morris water maze [3,4]. Thus, the intracisternal infusion of Aβ 1-42 into the lateral ventricle has been used to induce AD-like pathological and behavioral changes in animal model [3].

*Cistanche tubulosa* (Schenk) R. Wight (abbreviated as CT) is commonly used by traditional Chinese physicians to treat forgetfulness, impotence and senile constipation [5]. Recent studies have shown that pretreatment with total phenylethanoid glycosides of CT could improve the impairment of inhibitory avoidance response and neuronal apoptosis caused by quinolinic acid, Aβ 25-35, or common carotid artery ligation in mice [6-8] via its antioxidant activity and increasing the activities of intracellular antioxidant enzymes such superoxide dismutase and glutathione peroxidase [9]. Total phenylethanoid glycosides mainly include 2’-acetylacteoside, acteoside, cistanosides, echinacoside and isoacteoside [10]. Echinacoside and acteoside can prevent the memory impairment caused by scopolamine in mice [11,12]. Therefore, we hypothesized that CT may have beneficial effects in AD patients. To test this hypothesis, we used CT extract which the contents of phenylpropanoid glycosides were quantified to investigate the effects against amyloid-induced histopathological and behavioral changes in an AD-like rat model. According to a meta-analysis of Myhrer on four behavioral tasks [13], acetylcholinergic and catecholaminergic activities have a high influence on learning and memory. The AD-like rat model causes the dysfunction of central neurotransmitters such as acetylcholine and catecholamines, which is closely related to memory deficits [3,14]. Hence, we further demonstrated the role of central neurotransmitter function in CT extract-induced reversal of memory impairment caused by Aβ 1-42 infusion by measuring the levels of central neurotransmitters and the activities of related enzymes.

## Methods

### Preparation of plant extract

The dried materials of *Cistanche tubulosa* (Schenk) Wight (Citu970429) were identified by Development Center for Biotechnology (Taipei, Taiwan, R.O.C.) and deposited in Sinphar Tian-Li Pharmaceutical Co., Ltd. (Hangzhou, China). The aqueous extract of CT contains three phenylethanoid glycosides, echinacoside (25.4%), acteoside (3.8%), and isoacteoside (4.1%), which were quantified by high performance liquid chromatography plus photodiode-array detector according to the method of Jiang et al [9]. CT extract was freshly prepared with sterile distilled water.

### Subjects

Male Sprague-Dawley rats weighing 300-350 g, obtained from BioLASCO Taiwan Co. Ltd., were used in present study. They were randomly housed at a density of four rats per wire-mesh cage (39 × 26 × 21 cm) in a temperature-(23 ± 1°C) and humidity-(60%) regulated environment with free access to standard food in pellets and tap water and, on a 12 h - 12 h light/dark cycle (light phase: 08:00 to 20:00 h). The experimental protocol (Protocol No. 99-127-B) was approved by the Institutional Animal Care and Use Committee (IACUC) of China Medical University and the care of animal was carried out according to the Guiding Principles for the Care and Use of Laboratory Animals. After 1 week of acclimatization, rats were used for the below experiments.

### AD-like rat model

The AD-like rat model was developed by infusing Aβ 1-42 into the cerebral ventricle [4]. Briefly, rats were anesthetized with phenobarbital (45 mg/kg, i.p.) and positioned in a David Kopf stereotaxic instrument. An infusion cannula was implanted into the left cerebral ventricle (AP -1.5, ML +0.9, V -3.6 from bregma), and a continual infusion of Aβ 1-42 (300 pmol/day) was maintained for at least two weeks by attaching an infusion cannula to a mini-osmotic pump (Alzet 2002; Alza, Palo Alto, CA, USA).

### Experimental schedule

**Figure 1:**
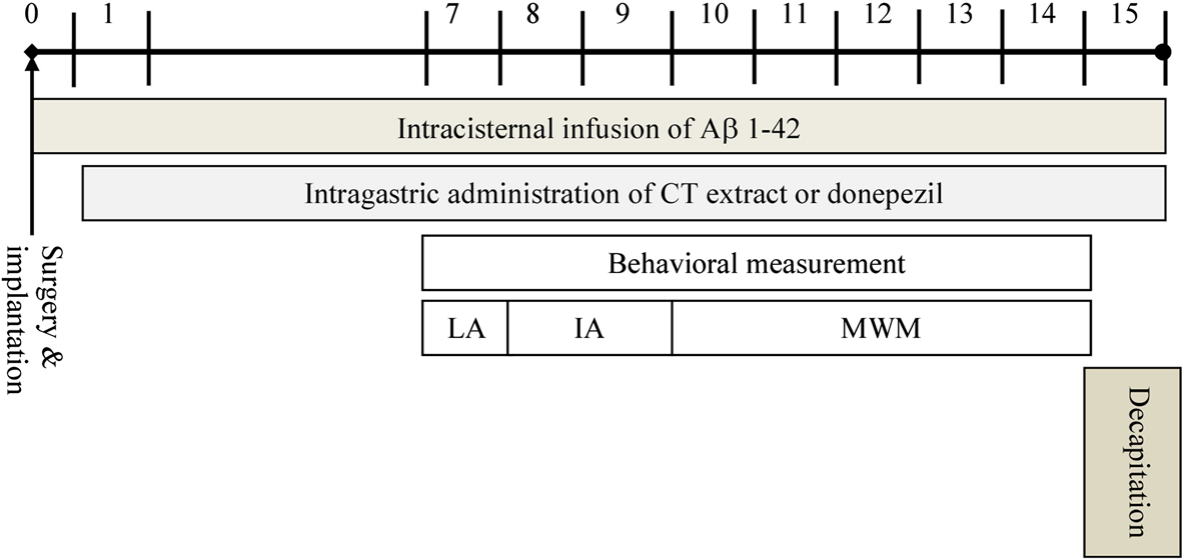
**Experimental schedule.** LA: Locomotor activity assay, IA: Inhibitory avoidance test, MWM: Morris water maze test.

After the implantation of the cannula and the attachment of the mini-osmotic pump, Aβ 1-42 infusion began, and this day was designated as day 0. On the next day (day 1), the rats were orally given with sterile distilled water, CT extract (100, 200 mg/kg) or donepezil (0.75 mg/kg) throughout Aβ 1-42 infusion period. CT extract (100, 200 mg/kg) or donepezil (0.75 mg/kg) was also still given to rats 1 h before the behavioral tests, including open field test and hole test (day 7 after Aβ 1-42 infusion), inhibitory avoidance test (day 8-9 after Aβ 1-42 infusion) and Morris water maze (day 10-14 after Aβ 1-42 infusion). On day 15 after Aβ 1-42 infusion, the rats were killed 1 h after the last administration of CT extract (100, 200 mg/kg) or donepezil (0.75 mg/kg) for the measurement of acetylcholinesterase (AChE) and monoamine oxidase (MAO) activities in the brain and the levels of central neurotransmitters and their metabolites. The schedule for surgery, drug treatment, and behavioral tests is shown in Figure 1.

### Locomotor and exploratory activities

Locomotor and exploratory activities were measured by an open-field task (Coulbourn Instruments L.L.C., PA, USA). Each animal was placed individually at the apparatus and observed for 10 min to record the movement time, length and velocity (locomotor activity), entries into the hole and time spent (exploratory activity) by TruScan software v 2.07 (Coulbourn Instruments L.L.C., PA, USA) [15].

### Inhibitory avoidance test

The inhibitory avoidance apparatus for rats (Coulbourn Instruments L.L.C., PA, USA) was used. The room was dark during the experimental sessions that were conducted between 09:00 and 12:00. During the training trial (day 8), each rat was placed in the light compartment with its back to the guillotine door and the time (step-through latency) was measured until the rat entered the dark compartment. After the rat entered the dark compartment, the door was closed. An inescapable foot shock (0.8 mA for 2 s) was delivered through the grid floor. The rat was removed from the dark compartment 5 s after the shock. Then, the rat was put back into its home cage until the retention trial. Twenty-four hours later, the retention trial (day 9) was conducted. The rat was again placed in the light compartment, the door was opened and the step-through latency was recorded as a measure of memory retention [15]. An upper cut-off time of 300 sec was set.

### Morris water maze task

For the assessment of spatial performance function, the rats were tested in a Morris water maze, which consisted of a black circular stainless pool (a diameter of 165 cm and a height of 60 cm), filled with 23 ± 1°C water to a depth of 35 cm. The maze was divided geographically into four equal quadrants and included release points in each quadrant. The position of white rat in the black pool was recorded by a video camera and an automated video tracking system device equipped with EthoVision XT software (Noldus Information Technology, Leesburg, VA, USA). The swim path and escape latency to find the platform were recorded for each trial. Each rat was given two trials per day for four consecutive days (day 10 - 13 after Aβ 1-42 infusion) to find the Plexiglass hidden platform (a diameter of 10 cm) that was situated in the center of the northeast quadrant and submerged 1.0 cm below the surface of the water. A trial was initiated by placing the rat in the water facing the pool wall in one of the four quadrants. For each trial, the rat was allowed to swim for maximum of 120 sec to find the platform. When successful, the rat was allowed a 30-sec rest period on the platform. If unsuccessful within the aborted time period, the rat was given a score of 120 sec and then physically placed on the platform and allowed the 30-sec rest period. In either case, the rat was immediately given the next trial after the rest period [15]. The day following four daily sessions (day 14), the probe test was performed as a measure of reference memory. The Plexiglass platform was removed from the pool, and each rat was released from the quadrant opposite to where the platform had been located. The parameters measured from probe test during 60 s were time and distance in each quadrant especial spent searching for the platform in the training quadrant, and swimming speed [15].

### Aβ 1-42 immunohistochemical stain

Rats were anesthetized with sodium pentobarbital (45 mg/kg) and perfused with saline through their left cardiac ventricle, followed by 4% paraformaldehyde in physiological saline. After postfixation, the whole brain was removed and prepared for paraffin slices. Three brain sections (10 μm) of each rat were obtained on a microtome (Leica 2030 Biocut). The tissues were incubated overnight at 4°C with a mouse anti-human amyloid β protein 17-24 monoclonal antibody (1:300 dilution, Dakopatts A/C; Glostrup, Denmark). Immunolabeled sections were developed with 0.05% diaminobenzidine using a Vectastain kit (Vector Laboratories, Burlingame, CA, USA). Brain sections were measured under 40 x magnification, and at least 20 fields from each brain section were counted by an image analyzer (Leica, Q500MC, Nussloch, Germany).

### Measurement of cortical and hippocampal neurotransmitter levels

For the measurement of acetylcholine levels, rats were sacrificed by microwave irradiation. For the measurement of catecholamine levels, rats were decapitated. Their brains were rapidly removed from the skull and immediately separated into the cortex and hippocampus on ice according to the protocol of Glowinski and Iversen [16]. Then, all brain tissues were homogenized with ice-cold 0.2 M perchloric acid and centrifuged at 14,000 rpm for 15 min at 4°C. The supernatants were collected, put into 0.22 μM Ultrafree MC centrifugal filter units (Millipore, USA) and again centrifuged for 5 min at 4°C. The collected samples were stored at -80°C for the measurement of the concentrations of central neurotransmitters and their metabolite by high performance liquid chromatography with electrochemical detection (EICOM HTEC-500, Japan).

### Determination of cortical and hippocampal AChE and MAO activity

Each cortex and hippocampus were homogenized in 9 vol ice-cold phosphate buffered saline. Homogenates were centrifuged at 12,000 rpm for 15 min at 4°C, and the supernatants were stored at –80°C until use. AChE activity was measured using Ellman method [17]. Brain homogenates were incubated with 5,5’-dithiobis(2-nitrobenzoic acid) at 25°C for 10 min and then acetylthiocholine was added for color development. The production of 5-thio-2-nitrobenzoic acid was measured at 412 nm. AChE activity was expressed as U AChE per mg protein. MAO-A and MAO-B activities were determined by incubating the reaction mixture including rat brain homogenates, 5 U/mL horseradish peroxidase, 100 μM amplex red, and the substrate (5 mM serotonin for MAO-A or 5 mM benzylamine for MAO-B) at 25°C for 60 min. The enzyme activity was expressed as the percentage of the activity relative to vehicle-infused rats [18]. The protein content was determined with a Bio-Rad protein assay kit using bovine serum albumin as a standard.

### Statistical analysis

The data of inhibitory avoidance test was analyzed using a Kruskal-Wallis non-parametric one-way analysis of variance, followed by Mann-Whitney’s *U*-test. The data from spatial performance, probe test, the ratio of amyloid deposition, the activities of AChE and MAO, and the levels of central neurotransmitters and their metabolites were subjected to a one-way analysis of variance (ANOVA) followed by Scheff’s test. The criterion for statistical significance was *P* < 0.05 in all statistical evaluations.

## Results

### Effects of CT or donepezil on locomotor and exploratory activities

**Figure 2:**
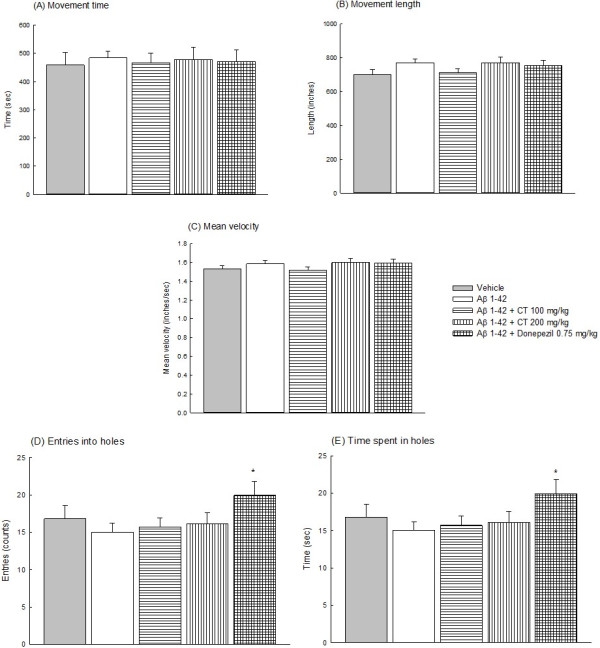
**Effects of CT extract (100, 200 mg/kg) or donepezil (0.75 mg/kg) on the locomotor activity including movement time (A), movement length (B) and mean velocity (C), and the exploratory behavior including entries into holes (D) and time spent in holes (E) in Aβ 1-42-infused rats.** Columns indicate means ± SEM (N = 20). *P < 0.05 compared with Aβ 1-42-infused rats.

There was no difference in movement time, length or velocity between vehicle-infused and Aβ 1-42-infused groups (*P > 0.05*). CT extract (100, 200 mg/kg) or donepezil (0.75 mg/kg) did not alter the movement time, length or velocity of Aβ 1-42-infused rats (*P* > 0.05) (Figure 2 (A) - (C)). There was slight decrease but not statistically significant in the time spent in the hole and entries into the hole between vehicle-infused and Aβ 1-42-infused group (*P* > 0.05). CT extract (100, 200 mg/kg) did not alter the time spent in the hole or entries into the hole of Aβ 1-42-infused rats (*P* > 0.05). However, donepezil at 0.75 mg/kg increased the time spent in the hole and the entry count into the hole of Aβ 1-42-infused rats (*P* < 0.05) (Figure 2 (D) and (E)).

### Effects of CT or donepezil on inhibitory avoidance task and morris water maze

**Figure 3:**
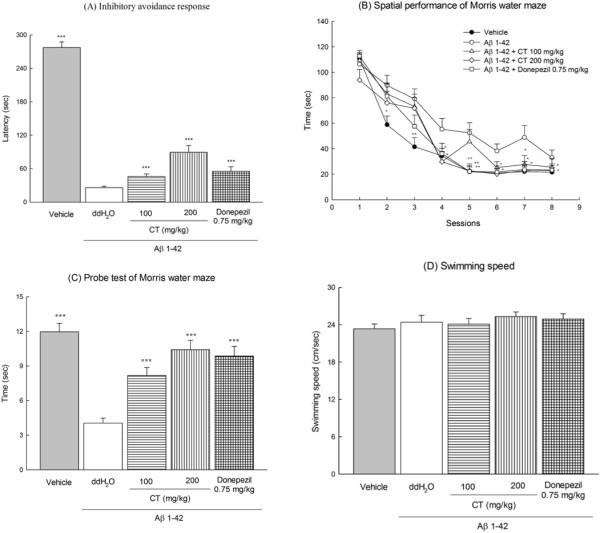
**Effects of CT extract (100, 200 mg/kg) or donepezil (0.75 mg/kg) on the step-through latency of the inhibitory avoidance task (A), the spatial performance (B), probe test (C), and swimming velocity (D) of the Morris water maze in Aβ 1-42-infused rats.** Columns indicate means ± SEM (N = 20). *P < 0.05, **P < 0.01, ***P < 0.001 compared with Aβ 1-42-infused rats.

In the acquisition trial of the inhibitory avoidance test, there was no difference in the step-through latency between any of the treatment groups (data not shown). However, a significant reduction in the step-through latency in the retention trial was observed in Aβ 1-42-infused groups compared to vehicle-infused group (*P* < 0.001). CT extract (100, 200 mg kg) or donepezil (0.75 mg/kg) increased the step-through latency of the retention trial in Aβ 1-42-infused rats (*P* < 0.001) (Figure 3 (A)).

The spatial performance over eight trials of four training days (from day 10 to day13 after Aβ 1-42 infusion), particularly trials 3 ~ 7, in Aβ 1-42-infused group was significantly impaired compared to that of vehicle-infused group (*P* < 0.01). CT extract (200 mg/kg) or donepezil (0.75 mg/kg) after Aβ 1-42 infusion ameliorated the impairment of spatial performance caused by Aβ 1-42 infusion (*P* < 0.05, *P* < 0.01) (Figure 3 (B)).

Compared to vehicle-infused group, Aβ 1-42-infused group showed a significant decrease in the time spent in the platform-quadrant during training (*P* < 0.001). All doses of CT extract and donepezil (0.75 mg/kg) significantly increased the time spent in the platform-quadrant relative to Aβ 1-42-infused group (*P* < 0.001) (Figure 3 (C)). In the swimming velocity, there were no differences between vehicle-infused group, Aβ 1-42-infused group, or CT extract or donepezil treatment groups (*P* > 0.05) (Figure 3 (D)).

### Effects of CT or donepezil on Aβ 1-42 deposition

**Figure 4:**
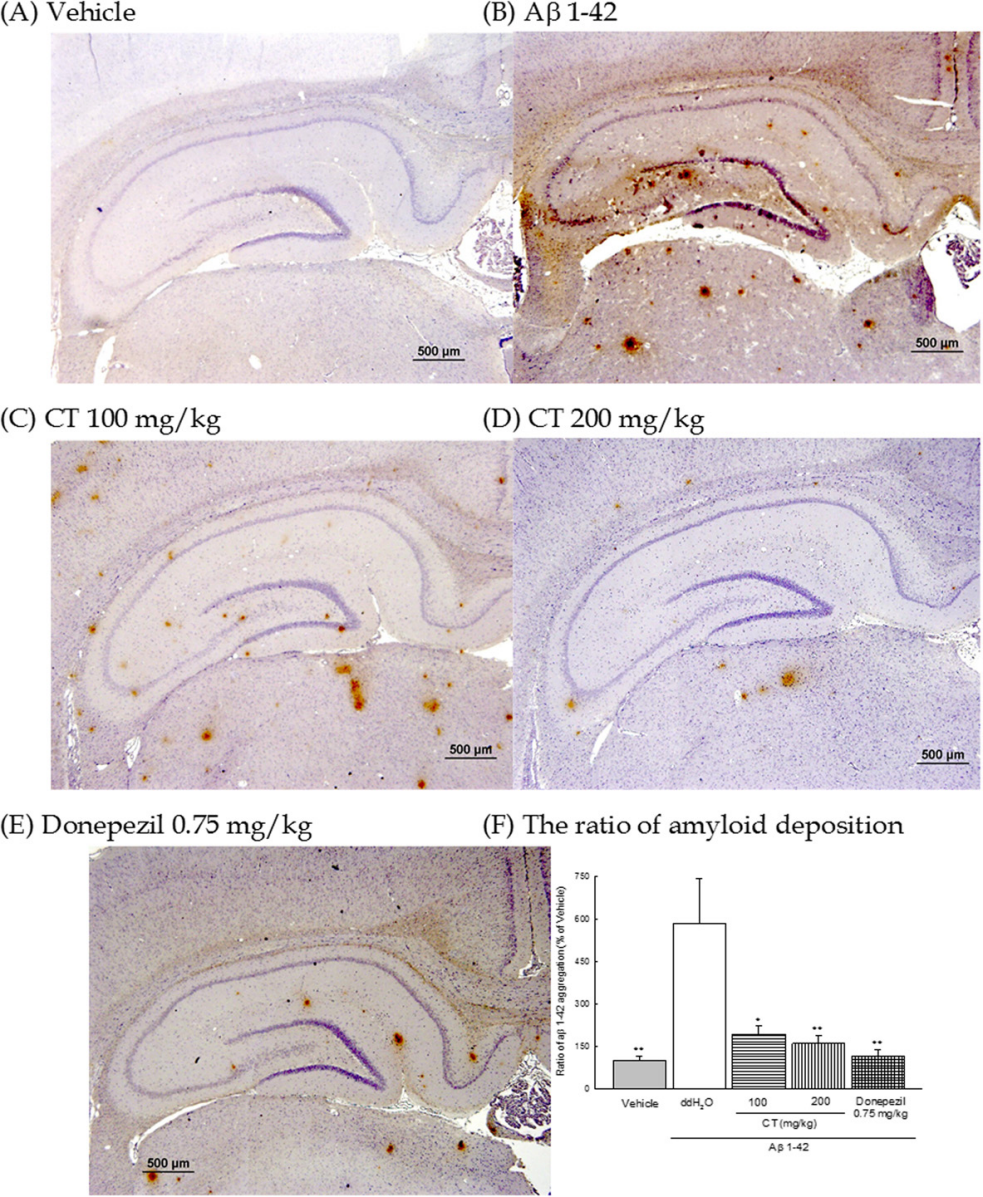
**Effects of CT extract (100, 200 mg/kg) or donepezil (0.75 mg/kg) on the Aβ 1-42 deposition in Aβ 1-42-infused rats. (A)** Vehicle-infused group, **(B)** Aβ 1-42-infused group, **(C)** CT extract (100 mg/kg)-treated group, **(D)** CT extract (200 mg/kg)-treated group, **(E)** donepezil (0.75 mg/kg)-treated group, **(F)** the ratio of amyloid depsotion. Columns indicate means ± SEM (N = 6). *P < 0.05, **P < 0.01 compared with Aβ 1-42-infused rats.

The deposition of amyloid β protein in the brains of Aβ 1-42-infused rats was more than that of vehicle-infused rats (Figure 4 (A) and (B)). CT extract (100, 200 mg/kg) or donepezil (0.75 mg/kg) decreased the deposition of amyloid β protein in the brains of Aβ 1-42-infused rats (Figure 4 (C) - (E)). The effects of CT extract or donepezil on the ratio of Aβ 1-42 deposition in the brains of Aβ 1-42-infused rats are shown in Figure 4 (F). Compared to vehicle-infused group, Aβ 1-42-infused group showed a significantly greater ratio of Aβ 1-42 deposition in the brain (*P* < 0.01). CT extract (100, 200 mg/kg) or donepezil (0.75 mg/kg) decreased the ratio of Aβ 1-42 deposition in the brain of Aβ 1-42-infused rats (*P* < 0.05, *P* < 0.01).

### Effects of CT or donepezil on brain neurotransmitters and their metabolites

Aβ 1-42 continual infusion decreased cortical and hippocampal acetylcholine levels (*P* < 0.05 for cortex, *P* < 0.001 for hippocampus), but only decreased hippocampal choline levels (*P* < 0.01). CT extract (200 mg/kg) or donepezil (0.75 mg/kg) reversed this decrease in cortical and hippocampal acetylcholine levels in Aβ 1-42-infused rats (*P* < 0.05) (Table 1).

**Table 1:** Effects of CT extract (100, 200 mg/kg) or donepezil (0.75 mg/kg) on the levels of cortical and hippocampal neurotransmitters and their metabolites in Aβ 1-42-infused rats

	**The levels in cortex (nmol/mg protein for ACh and choline, ng/g protein for other transmitters)**						
	**ACh**	**Choline**	**MHPG**	**NE**	**DOPAC**	**HVA**	**DA**
Vehicle	224.25 ± 25.55**	629.00 ± 98.35	26.35 ± 2.72	15.68 ± 0.76	16.75 ± 1.70*	3.71 ± 0.58	3.92 ± 0.44**
Aβ 1-42	141.35 ± 7.95	553.10 ± 31.55	23.27 ± 1.97	13.86 ± 1.29	11.66 ± 1.13	2.76 ± 0.43	2.33 ± 0.28
CT extract							
100 mg/kg	145.98 ± 18.78	716.40 ± 69.81	29.91 ± 4.91	15.94 ± 1.30	13.89 ± 3.03	4.29 ± 0.93	2.95 ± 0.65
200 mg/kg	181.49 ± 13.71*	693.45 ± 48.05	24.51 ± 4.16	10.31 ± 1.07	9.50 ± 2.10	2.98 ± 0.85	2.23 ± 0.33
Donepezil	168.32 ± 6.58*	467.26 ± 32.85	15.16 ± 2.51*	10.73 ± 2.33	10.49 ± 2.38	2.42 ± 0.78	3.10 ± 0.46
	The levels in hippocampus (nmol/mg protein for ACh and choline, ng/g protein for other transmitters)						
	ACh	Choline	MHPG	NE	DOPAC	HVA	DA
Vehicle	72.88 ± 8.64***	295.26 ± 18.06**	639.83 ± 53.38	63.64 ± 2.56*	5.87 ± 0.92	3.68 ± 0.41	5.36 ± 0.75***
Aβ 1-42	28.45 ± 2.62	173.10 ± 18.15	639.23 ± 68.26	52.67 ± 4.21	5.13 ± 0.80	3.39 ± 0.36	1.00 ± 0.21
CT extract							
100 mg/kg	43.48 ± 3.14	238.20 ± 23.91	785.42 ± 139.84	57.98 ± 10.69	4.98 ± 0.78	3.90 ± 0.61	2.40 ± 0.65*
200 mg/kg	50.44 ± 8.98*	234.69 ± 13.26	704.84 ± 59.68	63.21 ± 4.79	5.98 ± 1.90	3.78 ± 0.64	3.09 ± 1.27*
Donepezil	51.11 ± 9.54*	177.69 ± 46.80	455.27 ± 46.81*	32.80 ± 3.88***	3.19 ± 1.00	2.45 ± 0.33	1.33 ± 0.37

Columns indicate means ± SEM (N = 6). *P < 0.05, **P < 0.01, ***P < 0.001 compared with Aβ 1-42-infused rats.

Continuous infusion of Aβ 1-42 decreased cortical and hippocampal DA levels (*P* < 0.01), but only decreased hippocampal NE levels (*P* < 0.05). CT extract (100 and 200 mg/kg) only reversed the decrease in DA levels in the hippocampus of Aβ 1-42-infused rats (*P* < 0.05). Donepezil did not alter the decrease in hippocampal DA levels (*P* > 0.05), but exaggerated the decrease in hippocampal NE levels in Aβ 1-42-infused rats (*P* < 0.01) (Table 1).

### Effects of CT or donepezil on Brain AChE, MAO-A and MAO-B activity

**Figure 5:**
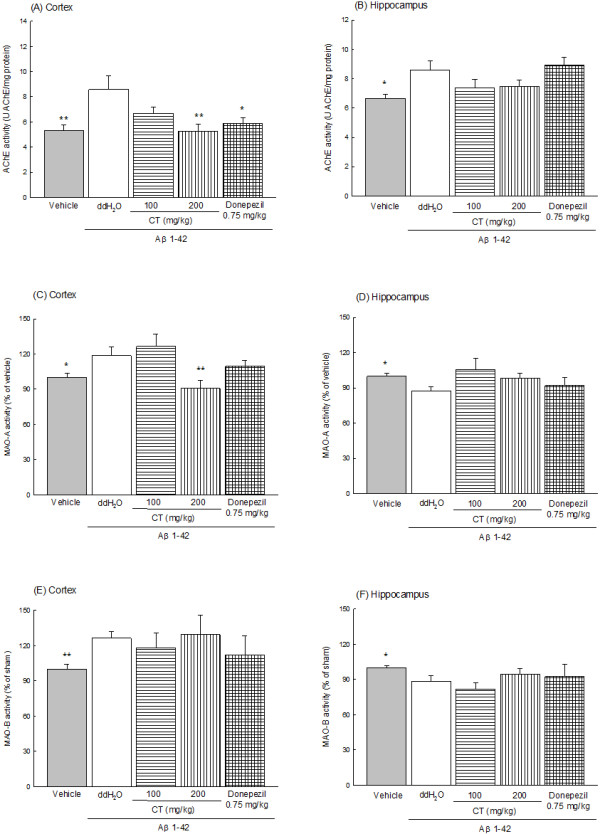
**Effects of CT extract (100, 200 mg/kg) or donepezil (0.75 mg/kg) on the AChE (A and B), MAO-A (C and D) and MAO-B (E and F) activity in the cortex (A, C and E) and hippocampus (B, D and F) of Aβ 1-42-infused rats.** Columns indicate means ± SEM (N = 6). *P < 0.05, **P < 0.01 compared with Aβ 1-42-infused rats.

Continuous infusion of Aβ 1-42 increased cortical and hippocampal AChE activities (*P* < 0.05 for hippocampus, *P* < 0.01 for cortex). CT extract (200 mg/kg) or donepezil (0.75 mg/kg) only reversed the increase in cortical AChE activity in Aβ 1-42-infused rats (*P* < 0.05 for donepezil, *P* < 0.01 for CT) (Figure 5 (A) and (B)).

Continuous infusion of Aβ 1-42 increased cortical MAO-A and MAO-B activity (Figure 5 (C) and (E), *P* < 0.05, *P* < 0.01), but decreased hippocampal MAO-A and MAO-B activities in the rats (Figure 5 (D) and (F), *P* < 0.05). CT extract at 200 mg/kg only reversed the increase in the cortical MAO-A activity in Aβ 1-42-infused rats (Figure 5 (C), *P* < 0.01). Donepezil (0.75 mg/kg) did not change the alteration of MAO-A and MAO-B activities in Aβ 1-42-infused rats (Figure 5 (C) - (F), *P* > 0.05).

## Discussion

Many reports from cell culture assays and animal experiments evidenced that Aβ is the key protein in AD patients [2,3]. Aβ 1-42 induced neuronal damage and apoptosis via oxidative stress *in vitro*[2,3]. Intracisternal injection of Aβ 1-42 into rats produced memory impairment, changes in brain morphology, and cholinergic neuronal degeneration [3,4]. In this study, the continual infusion of Aβ 1-42 to the cerebral ventricle by an osmotic pump (Alzet 2002) caused behavioral deficits including exploratory behavior, inhibitory avoidance response, and spatial performance of Morris water maze in rats. These symptoms were similar to previous reports [3,4]. Donepezil, a selective AChE inhibitor, possess neuroprotective activity against Aβ-induced toxicity i*n vitro* and *in vivo*[19,20]. Our present data are consistent with the above report [20] showing that donepezil ameliorated the behavior deficits caused by Aβ 1-42 infusion in rats. The intra-gastric administration of CT extract (100, 200 mg/kg) throughout Aβ 1-42 infusion period ameliorated the observed behavior deficits caused by Aβ 1-42. This memory-improving effect is consistent with other reports showing that total phenylethanoid glycosides of Cistanche species could protect the impairment of inhibitory avoidance response caused by an acute intracisternal injection of Aβ 25-35 in mice [6]. Recent clinical report also indicated that AD patients given with CT glycosides for 48 weeks showed no exacerbation of cognitive function [21]. Moreover, CT extract at used dosage (100, 200 mg/kg) contained echinacoside (25.4, 50.8 mg), acteoside (3.8, 7.6 mg) and isoacteoside (4.1, 8.2 mg) according to quantity assay performed in this study. Echinacoside (50 mg/kg) and acteoside (1 mg/kg) can prevent the memory impairments caused by scopolamine in mice [11,12]. Thus, we suggest that CT is a potential anti-amnesic and anti-dementia plant when it is quantified and standardized through its phenylethanoid glycosides, particularly echinacoside, acteoside and isoacteoside.

According to amyloid cascade hypothesis, memory deficits caused by Aβ are due to intracellular oxidative stress and neuronal apoptosis promoted by Aβ formation and subsequent aggregation and deposition [2,3]. Aβ deposition is closely associated with the dysfunction of central cholinergic neuronal circuits in the basal forebrain [2,3]. The dysfunction of central cholinergic neuronal systems, including decreased brain acetylcholine levels and an up-regulation of AChE activity, has been shown to be related to the degrees of amnesia and Aβ deposition in AD brain [2,3]. According to our above results, we further investigated the effects of CT extract or donepezil on Aβ-induced pathological changes in rats. We found that Aβ 1-42 continual infusion caused a major pathological change in Aβ deposition around hippocampus and prefrontal cortex and in a significant associated biochemical alteration: a decrease in acetylcholine and choline levels and an up-regulation of AChE activity particularly in hippocampal areas. These pathological symptoms were similar to other reports [4,22] showing that intracisternal injection of Aβ 1-42 over 2 weeks causes Aβ deposition in rat brain using immunohistochemical stain, and then causes an up-regulation in AChE activity within and around senile plaques. This up-regulation encourages the assembly of Aβ into fibrils, and these Aβ fibrils ultimately lead to Aβ neurotoxicity and particularly cholinergic dysfunction [23]. In Aβ 1-42-infused rats, donepezil decreased the ratio of Aβ deposition and the alteration in cortical AChE activity, and restored ACh levels in all brain areas. Our present data were consistent with another report [19,20] showing that donepezil might inhibit AChE activation to produce neuroprotective and memory-improving effects against Aβ 1-42. CT extract (100, 200 mg/kg) also decreased the deposition of Aβ, especially in hippocampal areas, but only 200 mg/kg dosage reversed the alterations in cortical AChE activity and the decrease in cortical and hippocampal acetylcholine levels. From these pathological and biochemical results, we suggested that CT extract at 200 mg/kg reversed cortical and hippocampal cholinergic function by decreasing the deposition of Aβ. Other reports indicated that total phenylethanoid glycosides of Cistanche species or acteoside could protect the memory deficits or neuronal damage caused by Aβ 25-35 through an antioxidant mechanism and decreasing the ratio of Bax/Bcl2 *in vivo* or *in vitro*[6,24]. Recent report further indicated that acteoside inhibited Aβ 1-42 aggregation in a dose-dependent manner by using the thioflavin-T assay [25]. Echinacoside decreased AChE activity in SAMP/8 mice [12]. Thus, we suggest that the memory-improving effects of CT extract might be related to decrease Aβ deposition and neurotoxicity, which leads to a reversal of negative changes in cortical and hippocampal cholinergic function.

Clinical researchers found that AD patients have complex neurochemical disturbances including the catecholaminergic, cholinergic and glutaminergic neuronal systems [26]. Some reports also found that AD patients have increased MAO-B activity compared to healthy controls, and this increased MAO-B activity might reflect abnormalities in the dopaminergic systems [27]. In AD-like rat model, Aβ 1-42 also causes catecholaminergic dysfunction [14]. Some nootropics or selegiline improved Aβ-induced memory deficits via enhancing catecholaminergic activity or modulating MAO activities [4,28]. In this study, Aβ 1-42 continual infusion also caused a decrease in cortical and hippocampal DA levels and in hippocampal NE levels. We also found that Aβ 1-42 continual infusion led to a differential alteration of cortical and hippocampal MAO activities, which included an up-regulation in cortical MAO-A and MAO-B activities and a downregulation in hippocampal MAO-A and MAO-B activities. These pathological symptoms of catecholaminergic dysfunction were similar to other reports [14,27]. Donepezil did not alter these catecholaminergic change caused by Aβ 1-42 infusion, but decreased hippocampal NE and MHPG levels compared to Aβ 1-42-infused rats This data is consistent with another report [19,20] showing that donepezil possesses memory-improving effects against Aβ 1-42 that act mainly through its selective AChE inhibiting effects but it possibly causes region-specific changes in some neurotransmitters. CT extract only reversed the decrease in hippocampal DA levels and the up-regulation of cortical MAO-A activity caused by Aβ 1-42 infusion. Hence, we suggest that CT extract at 200 mg/kg reversed the negative effects on hippocampal dopaminergic function mainly by decreasing the deposition of Aβ in the hippocampal areas. However, donepezil also decreased the deposition of Aβ, but did not affect the hippocampal dopaminergic function.

## Conclusion

CT is a potential anti-amnesic and anti-dementia plant based on our present data and other reports [6-8,21]. The contents of echinacoside and acteoside in this used dosage of CT extract is sufficient to produce memory-improving effects in SAMP/8 mice or scopolamine-induced amnesia [11,12]. Hence, we further suggest that, as an anti-dementia plant, it is important to standardize the content range of echinacoside, acteoside and isoacteoside in CT extract. Furthermore, the memory-improving mechanism of CT extract, which is different from that of donepezil that it decreased Aβ deposition and cholinergic dysfunction by inhibiting AChE activation and activating nicotinic receptors and phosphoinositide 3-kinase/Akt pathways [19,20], is due to the decrease in Aβ deposition and reversal of the cholinergic and hippocampal dopaminergic dysfunction. Moreover, other reports have indicated that total phenylethanoid glycosides of the Cistanche species possess neuroprotective and memory-enhancing effects through their antioxidant and neurotrophic activities [9,29,30]. Echinacoside and acteoside also possess neuroprotective effects in H_2_O_2_- MPTP- or Aβ 25-35-induced neurotoxicity via antioxidant and neurotrophic activities [24,31,32]. Hence, whether the protective effect of CT extract in AD-like rat model acts through anti-aggregating, neuroprotective or neurotrophic effects against Aβ should be investigated in the future.

## Abbreviations

Aβ 1-42: Amyloid β peptide 1-42; AChE: Acetylcholinesterase; AD: Alzheimer’s disease; CT: *Cistanche tubulosa* (Schenk) R. Wight; MAO: Monoamine oxidase.

## Competing interests

The authors declare that they have no competing interests.

## Authors’ contributions

CRW and MHS carried out all the experimentation, acquisition of data, conceived, designed, supervised the study, drafted and revised the manuscript. HCL identified the plants, and provided the plant extracts. All authors read and approved the final manuscript.

## Pre-publication history

The pre-publication history for this paper can be accessed here:

http://www.biomedcentral.com/1472-6882/14/202/prepub
